# Fine definition of the pedigree haplotypes of closely related rice cultivars by means of genome-wide discovery of single-nucleotide polymorphisms

**DOI:** 10.1186/1471-2164-11-267

**Published:** 2010-04-27

**Authors:** Toshio Yamamoto, Hideki Nagasaki, Jun-ichi Yonemaru, Kaworu Ebana, Maiko Nakajima, Taeko Shibaya, Masahiro Yano

**Affiliations:** 1 QTL Genomics Research Center, National Institute of Agrobiological Sciences, Kannondai 2-1-2, Tsukuba, Ibaraki 305-8602, Japan

## Abstract

**Background:**

To create useful gene combinations in crop breeding, it is necessary to clarify the dynamics of the genome composition created by breeding practices. A large quantity of single-nucleotide polymorphism (SNP) data is required to permit discrimination of chromosome segments among modern cultivars, which are genetically related. Here, we used a high-throughput sequencer to conduct whole-genome sequencing of an elite Japanese rice cultivar, Koshihikari, which is closely related to Nipponbare, whose genome sequencing has been completed. Then we designed a high-throughput typing array based on the SNP information by comparison of the two sequences. Finally, we applied this array to analyze historical representative rice cultivars to understand the dynamics of their genome composition.

**Results:**

The total 5.89-Gb sequence for Koshihikari, equivalent to 15.7× the entire rice genome, was mapped using the Pseudomolecules 4.0 database for Nipponbare. The resultant Koshihikari genome sequence corresponded to 80.1% of the Nipponbare sequence and led to the identification of 67 051 SNPs. A high-throughput typing array consisting of 1917 SNP sites distributed throughout the genome was designed to genotype 151 representative Japanese cultivars that have been grown during the past 150 years. We could identify the ancestral origin of the pedigree haplotypes in 60.9% of the Koshihikari genome and 18 consensus haplotype blocks which are inherited from traditional landraces to current improved varieties. Moreover, it was predicted that modern breeding practices have generally decreased genetic diversity

**Conclusions:**

Detection of genome-wide SNPs by both high-throughput sequencer and typing array made it possible to evaluate genomic composition of genetically related rice varieties. With the aid of their pedigree information, we clarified the dynamics of chromosome recombination during the historical rice breeding process. We also found several genomic regions decreasing genetic diversity which might be caused by a recent human selection in rice breeding. The definition of pedigree haplotypes by means of genome-wide SNPs will facilitate next-generation breeding of rice and other crops.

## Background

As molecular genetics technology advances, marker-assisted selection and quantitative trait locus (QTL) analysis are being routinely performed in crop genetics research. Consequently, results such as the isolation of key genes and molecular breeding have been achieved in several species, including rice [1-3]. Although the use of DNA markers has become a powerful tool for crop breeding, the approach has problems that must be resolved, such as the extremely low levels of DNA polymorphism that are often found among closely related rice cultivars, which has made it difficult to use DNA markers in genetic analysis. For example, several types of DNA markers with high levels of polymorphism between *indica *and *japonica *cultivars have been reported, but the level of polymorphism within *japonica *is low [4-7]. However, despite this low level of DNA polymorphism, wide phenotypic diversity is believed to exist [8]. This diversity is the major source of variation that has been targeted in breeding programs for rice improvement. Therefore, more robust methods to detect sequence polymorphisms among *japonica *accessions must be developed to help breeders and researchers understand the source of the phenotypic variations.

Single nucleotide polymorphisms (SNPs) are the most frequent polymorphisms in the genomes of most organisms. Since the rice genome was recently sequenced with high accuracy using a *japonica *cultivar, Nipponbare [9], comprehensive detection of SNPs using the Nipponbare sequence as a reference has become an effective tool. Thus far, whole-genome detection of SNPs has been carried out by comparisons between Nipponbare and an *indica *line, 93-11 [10,11]. However, the degree of polymorphism in SNP markers is still low within temperate *japonica *cultivars [12,13]. This has been caused by a shortage of an absolute quantity of sequences to be compared. Recently, high-throughput sequencers such as the GS FLX system (Roche Applied Science, Mannheim, Germany), the Solexa Genome Analyzer (Illumina Inc., San Diego, CA, USA), and the SOLiD system (Applied Biosystems, Foster City, CA, USA) have enabled researchers to produce sequences of more than 100 Mb and as high as several Gb per run [14,15], and have thus been used for resequencing, transcriptome, and epigenetic analysis, among other analyses (reviewed by [16,17]). In fact, the detection of genome-wide SNPs in eukaryotes using high-throughput resequencing has been reported for the human, nematode, Arabidopsis, and rice genomes [18-21].

The process of crossing and selection during plant breeding has resulted in the recombination and shuffling of ancestral chromosome blocks (pedigree haplotypes) to create new variation. The identification of such haplotypes in developed cultivars provides valuable information to support further improvement of rice and other crops. Researchers have attempted to define such haplotypes in rice using several types of DNA markers [22], but owing to technical limitations, fine-scale definition of pedigree haplotypes based on a large number of DNA markers has not yet been performed. In this context, SNPs identified by means of high-throughput genotyping based on array technology could be a useful tool [23,24]. Such large-scale, genome-wide SNP typing in barley has revealed the genetic constitution of old landraces and modern cultivars [25]. Two independent technological innovations, the high-throughput sequencer and hybridization arrays, have provided a new opportunity to determine the historical flow of pedigree haplotypes during the breeding of rice cultivars.

In this study, we attempted to identify genome-wide SNPs in closely related rice cultivars. To do so, we first completed a whole-genome sequence of a temperate *japonica *rice cultivar, Koshihikari, which is both the most popular cultivar in Japan and closely related to Nipponbare. After *in silico *mapping of numerous consensus reads against the Pseudomolecules for Nipponbare, we carried out comprehensive detection of genome-wide SNPs between the two cultivars. In addition, we applied an array-based SNP detection system to 151 representative Japanese *japonica *rice cultivars, and were able to define haplotype blocks and trace their inheritance during rice breeding in Japan, even though these cultivars were closely related. These results revealed the changes in genetic diversity in particular chromosomal regions during the long history of rice breeding in Japan. This information and the tools developed will provide new opportunities to establish selection criteria for the breeding of rice and other crop species.

## Results

### *In silico *mapping of the Solexa reads to the reference rice genome

**Figure 1:**
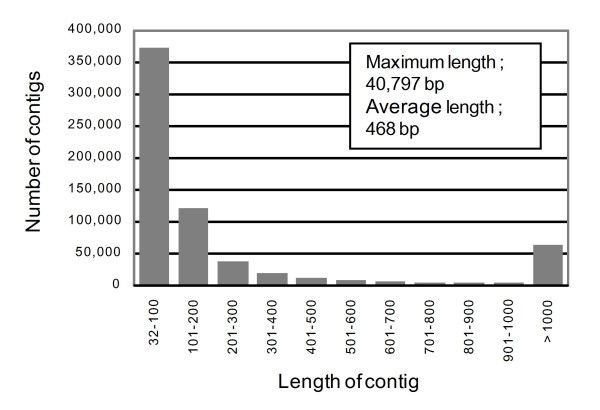
**Frequency distribution of contig lengths among the Koshihikari reads**. The contig lengths represent consensus sequences of the Koshihikari reads mapped to the Nipponbare genome.

We mapped a large number of short reads of the Koshihikari genome onto the Nipponbare genome and assembled them into a consensus sequence (Table 1). A total of 179 296 448 uniquely mapped Koshihikari reads corresponded to 5 890 906 099 bp (5.9 Gb henceforth) of the genome (Table 1). The sequence depth of the Koshihikari reads varied from 14.1× the genome on chromosome 12 to 18.3× on chromosome 2, and averaged 15.7× across the genome. The mapped reads were almost equally distributed (additional file 1), and covered 306 177 972 bp (approximately 80.1%) of 382 150 945 bp of the Nipponbare genome [9]. The Koshihikari consensus genome was composed of 654 543 contigs, with lengths ranging from 32 to 40 797 bp, and averaging 468 bp. Contigs shorter than 100 bp accounted for approximately 56.9% of the total (Figure 1). About 76.0 Mb of the Nipponbare genome was not covered by the short Koshihikari reads. These sequences could be classified into three main categories: 64.3 Mb (84.6%) comprised retrotransposons or short duplicated repeats, 10.1 Mb (13.3%) were regions in which undefined bases (Ns) dominated in the Nipponbare reference genome, and 766 kb (1.2%) were regions showing homology with the chloroplast and mitochondrial genomes. In addition, 647 kb (0.85%) were sequences for which Koshihikari short reads could not be mapped, probably due to multiple SNPs and insertion-deletions (InDels).

**Table 1:** Coverage of the Nipponbare Pseudomolecules 4.0 database by the Koshihikari reads from the Solexa Genome Analyzer.

				Uniquely mapped reads				
							
Chromosome	Pseudomolecule of Nipponbare (bp)^a^	Koshihikari aligned length (bp)^b^	Coverage (%)	Total number	bp	Total number of mapped contigs	Sequencing depth (fold)	Total of SNPs
1	45,064,769	36,886,997	81.9	21,887,208	719,138,056	63,889	16.6	10,980
2	36,823,111	31,152,504	84.6	19,972,645	656,243,813	53,822	18.3	4,113
3	37,257,345	32,175,988	86.4	19,309,856	634,463,329	51,236	17.5	2,773
4	35,863,200	27,331,878	76.2	15,718,358	516,454,041	69,662	14.6	9,891
5	30,039,014	24,458,877	81.4	14,277,427	469,092,491	51,892	15.7	1,032
6	32,124,789	25,763,900	80.2	14,992,102	492,566,411	53,317	15.8	1,165
7	30,357,780	24,230,102	79.8	13,935,933	457,866,127	55,090	15.4	7,561
8	28,530,027	22,970,652	80.5	13,106,335	430,601,662	53,667	15.2	6,426
9	23,843,360	19,060,074	79.9	11,008,304	361,678,832	41,137	15.7	178
10	23,661,561	18,592,212	78.6	10,572,960	347,371,203	43,569	15.3	3,617
11	30,828,668	22,439,806	72.8	12,582,930	413,402,408	58,282	14.5	12,216
12	27,757,321	21,114,982	76.1	11,932,390	392,027,726	58,990	14.1	7,099

Total or average^c^	382,150,945	306,177,972	80.1^d^	179,296,448	5,890,906,099	654,543	15.7	67,051

^a ^IRGSP, pseudomolecule build4

^b^Total length of aligned base except for unidentified base 'N'.

^c^Total number except the column of coverage and sequencing depth, these are discribed as average value.

^d^Coverage is calcurated based on Koshihikari aligned length/Pseudomolecule of Nipponbare. The value is slightly different from average of coverage of respective chromosome.

### Detection and distribution of SNPs between Koshihikari and Nipponbare

**Figure 2:**
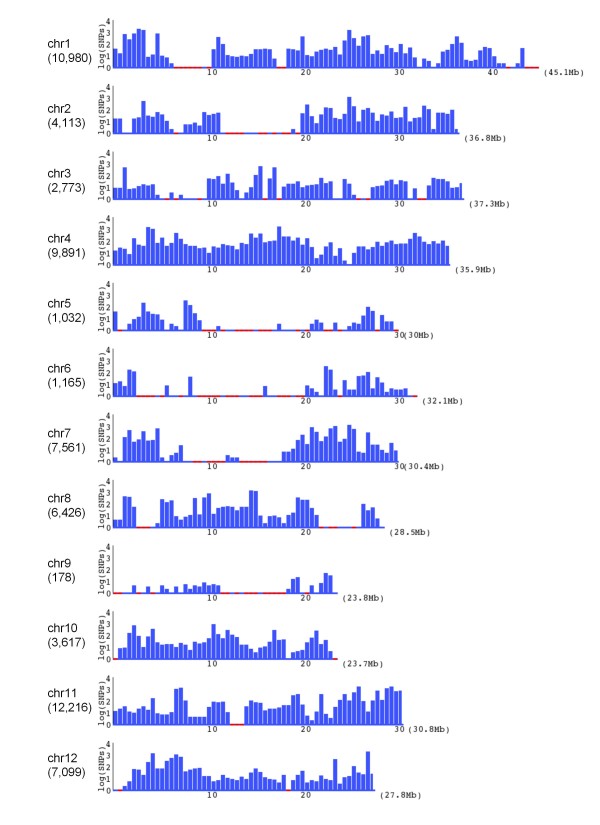
**Distribution of SNPs between Koshihikari and Nipponbare in the 12 rice chromosomes**. The number of SNPs in each chromosome is shown in brackets. The *x*-axis represents the physical distance along each chromosome, split into 500-kb windows. The orange lines represent regions in which no SNPs were detected. The *y*-axis indicates the common logarithm of the number of SNPs.

We detected a total of 67 051 SNPs between Koshihikari and Nipponbare (Table 1; http://koshigenome.dna.affrc.go.jp/). Although the average SNP density was 1 per 5.7 kb, the density varied among the chromosomes (Table 1), from 1/134.0 kb (178 SNPs) on chromosome 9 to 1/2.5 kb (12 216 SNPs) on chromosome 11. Moreover, the distributions of the SNPs were uneven within a chromosome (Figure 2); for example, there were 17 high-density regions with >0.5 SNPs/kb, including 1853 SNPs in 2.5-3 Mb on chromosome 1, and 1745 SNPs in 27-27.5 Mb on chromosome 12. We randomly selected 64 SNPs from all chromosomes for validation. Of these, we confirmed 63 SNPs using the traditional Sanger method using a capillary sequencer, indicating a high level of reliability in SNP detection.

To determine the optimal sampling effort by means of high-throughput sequencing to obtain as many genome-wide SNPs as possible, we simulated the relationships among sequencing depth, genome coverage, and number of SNPs detected (additional file 2; see Methods for details). As the sequence depth increased to 10× the genome, both the genome coverage and the number of SNPs detected increased. However, although the number of SNPs increased as the sequencing depth increased to 15×, genome coverage did not improve. This suggests that a 10× sequencing depth should be sufficient to detect SNPs distributed throughout the genome for alignment based on short reads.

A SNP in the coding sequence for a gene occasionally causes non-synonymous amino acid substitution, leading to a decrease or loss of functional activity of the transcripts. We aligned the detected SNPs with the annotated RAP2 Nipponbare full-length cDNA database [26] to investigate whether these SNPs might affect the function of the gene products. Of 3352 SNPs that occurred in 1077 genes, 1800 SNPs (53.7% of the total) in 794 genes (73.7% of the total) were non-synonymous, whereas 1552 SNPs (46.3%) in 594 genes (55.2%) were synonymous, and 311 genes (28.9%) included both synonymous and non-synonymous changes. It also appears that 18 and 7 SNPs, respectively, resulted in internal termination codons in the Koshihikari and Nipponbare alleles. This data is available at http://koshigenome.dna.affrc.go.jp/.

### Definition of genome-wide pedigree haplotypes of the Japanese rice cultivars by means of SNP array analysis

We selected 151 representative rice cultivars that have been grown during the past 150 years from Japanese landrace and cultivar collections (additional file 3). Since these cultivars are breeding lines and are therefore not appropriate for biological classification such as by population structure analysis (additional file 4), we roughly classified them into three groups based on the year in which they were developed. Group 1 consists of 38 landraces and two cultivars that were grown from the late 1800s to about 1920. Group 2 includes 49 cultivars that were developed from 1931 to 1974, when high yield was the major objective of breeding programs. The last 64 cultivars (Group 3) are recent cultivars that were developed from 1975 to 2005, when the breeding objective changed from high yield to good eating quality. By processing the genotype data for 1917 SNPs (see Methods), we defined the genome compositions of all the cultivars based on whether the alleles were similar to those of Koshihikari (No. *61 *in additional file 3), Nipponbare (*72*), or neither (additional file 5). The frequencies of the Koshihikari type tended to increase in most of the recent cultivars (Group 3) compared with older cultivars. All the cultivars could be distinguished from the others except for the pair of Fujiminori (*66*) and Reimei (*75*), the latter of which was developed by means of gamma-irradiation mutagenesis of the former.

**Figure 3:**
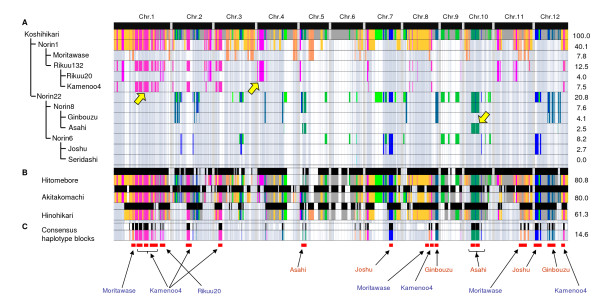
**Patterns of the pedigree haplotype blocks of Koshihikari and its related cultivars**. Only haplotype blocks longer than 500-kb of Koshihikari (No. *61 *in additional file 3) and consensus haplotype blocks among three progeny cultivars, Hitomebore (*117*), Akitakomachi (*100*), and Hinohikari (*113*) are shown. The black bars at the top indicate the range of the blocks in the 12 rice chromosomes. Vertical gray lines represent the borders between chromosomes. The numbers at the right indicate the proportion of the Koshihikari genome accounted for by the haplotype blocks. *(A) *Patterns of haplotype blocks in 12 parental cultivars in the pedigree chart of Koshihikari. Five warm colors (the red component of the 24-bit RGB color equaled 255 for all colors) indicate that the haplotype blocks were derived from the paternal parent, Norin1 (No. *39*). Seven cool colors (the red component of the 24-bit RGB color equaled 0 for all colors) indicate that the haplotype blocks were derived from the maternal parent, Norin22 (*47*). Gray indicates unidentified haplotype blocks that may have been derived from either parent. The three yellow arrows indicate pedigree haplotypes that inherited more than 2 Mb of their length with a density of more than 1 SNP/100 kb. *(B) *The haplotype blocks of Koshihikari in three progeny cultivars, Hitomebore (*117*), Akitakomachi (*100*), and Hinohikari (*113*). *(C) *Consensus haplotype blocks between Koshihikari and the three progeny cultivars. Only blocks derived from the six ancestral cultivars of Koshihikari (purple and red names) are indicated. Red horizontal bars represent consensus haplotype blocks longer than 1 Mb and the names of the ancestral landraces.

Figure 3A presents the estimated genome composition of Koshihikari based on the SNP and pedigree information (additional file 6). Although the reliability of the pedigree haplotype depends on the number of SNPs in the respective haplotype blocks, we could identify the ancestral origin of the pedigree haplotypes in 60.9% of the Koshihikari genome. This revealed that Koshihikari consists of haplotype blocks derived from six Japanese landraces: at least 7.8% of the total genome was derived from Moritawase (No. *29 *in additional file 3), 4.0% from Rikuu20 (*37*), 7.5% from Kamenoo4 (*31*), 4.1% from Ginbouzu (*23*), 2.5% from Asahi (*24*), and 2.7% from Joshu (*10*). Blocks longer than 2 Mb were inherited from Kamenoo4 (24.4-26.6-Mb region on chromosome 1), Rikuu20 (1.6-4.5-Mb region on chromosome 4), and Asahi (9.0-11.9-Mb region on chromosome 10), which are indicated by the yellow arrows in Figure 3A.

Hitomebore (No. *117*), Akitakomachi (*100*), and Hinohikari (*113*) are the second, third, and fourth most-grown cultivars in Japan, and were developed by crossing with Koshihikari (*61*), as shown in additional file 6. Figure 3B shows the genomic segments inherited from Koshihikari in these three cultivars. The proportions of the Koshihikari genome present in these three varieties were estimated to be 80.8% in Hitomebore, 80.0% in Akitakomachi, and 61.3% in Hinohikari. In total, 52 regions of conserved haplotype blocks, accounting for about 14.6% of the total genome, were also observed among the four cultivars (Figure 3C). In particular, 18 consensus haplotype blocks longer than 1 Mb from six landraces were predicted (red bars at the bottom of Figure 3C).

### Changes in genome diversity during modern rice breeding

We used the number of haplotypes per unit of genomic region estimated by the sliding-window approach as an index of genome diversity. The window size is ideally considered to be the same as the length of the haplotype block estimated by linkage disequilibrium (LD). We calculated that the LD decay was 2 Mb (additional file 7), which was higher than previously reported values in rice [27]. We suggest two reasons: (1) The breeding population has been highly structured by strong selection pressure. (2) Japanese cultivars with extremely high genetic similarity share segments inherited from the same ancestor. So we simulated the number of haplotypes by changing the window size around 10-SNPs, which was equivalent to 2 Mb (additional file 8). The changes in the number of haplotypes showed distinct differences among the three groups of cultivars, suggesting that a 10-SNP window is appropriate. However, since a small number of SNPs led to a long 10-SNP window (15.87 Mb) on chromosome 9 (additional file 9), we excluded this chromosome from further analysis. The mean physical length of the 10-SNP window ranged from 1.44 Mb on chromosome 1 to 6.00 Mb on chromosome 5.

**Figure 4:**
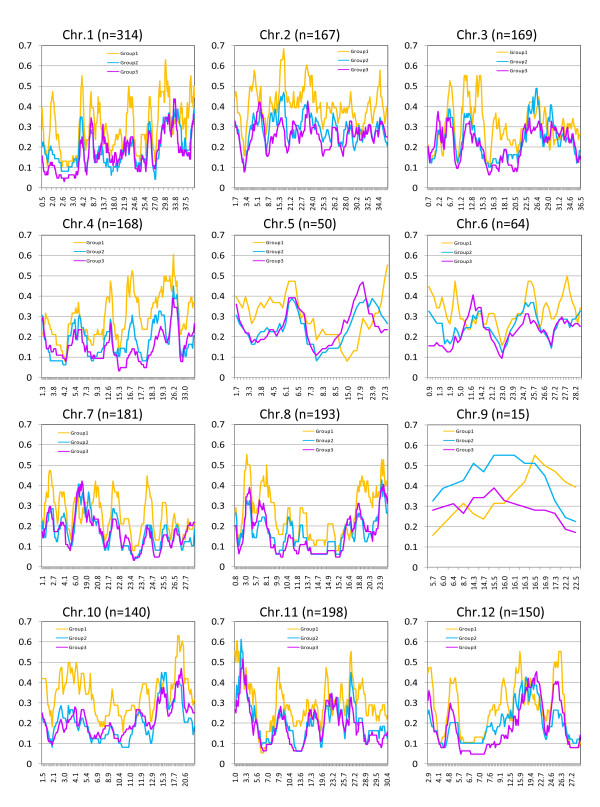
**Haplotype diversity index values for the 12 rice chromosomes in Japanese landraces and modern cultivars**. The diversity index was calculated on the basis of a 10-SNP window (see Methods for details). The number of haplotype windows (*n*) is indicated in parentheses. The *x*-axis shows the position between the start and end of the haplotype window. The *y*-axis shows the haplotype diversity index, which is calculated as the number of haplotypes divided by the number of cultivars in each group. The details of Groups 1, 2, and 3 are described in the Results and in additional file 3.

The total number of haplotypes on chromosome 2 (0.31/cultivar) was significantly higher than on the other chromosomes (Figure 4 and additional file 9). The highest densities were observed in regions of 19.5 Mb on chromosome 10 (0.49) and in 2.2 Mb on chromosome 11 (0.54). On the other hand, small regions with relatively low haplotype number (<0.1) were also observed, including regions of 2.6 Mb on chromosome 1, 4.2 Mb on chromosome 4, 23.5 Mb on chromosome 7, 15.2 Mb on chromosome 8, 6.9 Mb on chromosome 11, and 6.8 Mb on chromosome 12.

Groups 2 and 3 tended to have fewer haplotypes than Group 1 at either the entire chromosome level (additional file 9) or the chromosome-region level. Most of the regions showed a decreased number of haplotypes in Group 2, but several regions showed decreases in both Group 2 and Group 3, such as in regions of 0.9-1.3 Mb on chromosome 6 and 7.6-12.5 Mb on chromosome 12. However, we also observed regions where haplotype numbers increased from Group 1 to Group 3. Of these, the number of haplotypes in three chromosomal regions (8.5-23.9 Mb on chromosome 5, 11.6-14.2 Mb on chromosome 6, and 6.0-19.0 Mb on chromosome 7) might not be reliable because of the low SNP density. However, those in three regions (29.8-33.8 Mb on chromosome 1, 0.7-2.2 Mb on chromosome 3, and 19.4-22.7 Mb on chromosome 12) are reliable because they were based on a high SNP density.

## Discussion

### Whole-genome resequencing for comprehensive SNP detection

Ossowski et al. [20] reported that 87% of the total Arabidopsis reference genome was covered by Solexa reads. However, no similar study of rice had been reported. We found that 80.1% of the rice reference genome (Nipponbare) was covered by Solexa reads derived from a closely related cultivar, Koshihikari (Table 1). Our preliminary *in silico *mapping indicated that 79.9% of the 35-bp split-genome sequence of Nipponbare chromosome 1 was uniquely mapped to the corresponding region of the Nipponbare genome (data not shown), showing good agreement with the genome coverage. Despite the high level of genome coverage, 372 435 contigs (56.9%) were less than 100 bp long (Figure 1); this is unavoidable because of the short reads generated by the high-throughput sequencer. The lack of coverage of some regions was caused by large changes in chromosomal structure and therefore loss of contiguousness, but most omissions were due to unmapped reads with multiple SNPs, conserved domains, repeat sequences, or small InDels, which must be filled in after future advances in sequencing technology.

Though we mapped the Solexa reads at an average sequencing depth of 15.7×, the depth varied among genomic regions. Small chromosomal regions that were not covered well by the Koshihikari reads were found on all chromosomes (additional file 1). A region with lower depth that measured 6.8 to 7.1 Mb on chromosome 8 was consistent with a cluster of retrotransposons (data not shown). However, we could not fully explain the lower depth of the small chromosomal regions. Some cases might be explained by heterochromatic and repetitive regions [28] or by chromosome segment duplication [29]. The relationship between sequencing depth and efficacy in the comprehensive detection of SNPs is a key concern from the perspective of cost-effectiveness. Although the optimal sequencing depth depends on the study objectives, our data suggest that a sequencing depth of at least 10× would be necessary to discover genome-wide SNPs for use in haplotype analysis (additional file 2). Smith et al. [30] reported that the redundancy resulting from increasing the sequencing depth from 10× to 15× permits accurate and cost-effective detection of polymorphisms using the Solexa analyzer. Our results clearly support their conclusion.

### Distributions of the detected SNPs between closely related rice cultivars

We detected 67 051 SNPs between Koshihikari and Nipponbare (Table 1), with an average density of 5.7 kb/SNP. In previous comparisons between *indica *and *japonica*, the densities were 0.27 kb/SNP in a 2.4-Mb region on chromosome 4 between GLA4 and Nipponbare [31], and 0.93 kb/SNP across the whole genome between 93-11 and Nipponbare [11]. The density of SNPs between the Japanese cultivars in the present study was 1/6th to 1/21st those in the previous reports, which may reflect sequence divergence among the varietal combinations used in the analysis. Recently, a genome-wide resequencing analysis of 20 diverse *indica *and *japonica *cultivars [21] demonstrated that the average SNP density between two temperate *japonica *cultivars, Nipponbare and M202, was 25.1 kb/SNP (about 117 Mb/4662 SNPs). Monna et al. [12] reported a diversity analysis among nine rice accessions, including Koshihikari and Nipponbare, by means of Sanger sequencing of 1117 intergenic regions. Of these, 78 sites showed polymorphism between the two cultivars, and were distributed unevenly throughout the genome. We detected a large number of SNPs in regions that did not show any polymorphism, indicating the potential power of the present approach for SNP discovery even among closely related rice cultivars. However, we still identified extremely low numbers of SNPs in several chromosomal regions, such as in 6.5 to 9.5 Mb on chromosome 1, in 12 to 14 Mb on chromosome 2, and in 2.5 to 3 Mb on chromosome 8 (Figure 2). It is very likely that these chromosomal regions are conserved between Koshihikari and Nipponbare, because they share a common ancestral cultivar (Norin22), and thus probably share a common chromosomal segment. This would make these regions unsuitable sources of SNPs for use as DNA markers. To overcome the uneven distribution of SNPs, we are collecting additional SNP sets to fill the gaps by whole-genome sequencing of Japanese cultivars distantly related to Koshihikari and Nipponbare. On the other hand, Wang et al. [32] defined numerous genomic regions with low frequency of SNPs among rice cultivars as "SNP deserts", and suggested that they might be a result of rice domestication. Some of the regions with low frequencies of SNPs, such as on the distal end of the long arm of chromosome 1, at 10-12 Mb on chromosome 2 and at 10-15 Mb on chromosome 5 (Figure 2), overlap with these deserts, and may be legacies of domestication.

### Definition of the pedigree haplotype

Genome-wide SNP typing has been a powerful method for revealing the genomic constitutions of closely related lines or cultivars [25]. Huang et al. [33] proposed a new method of high-throughput genotyping and estimation of recombination points based on whole-genome resequencing of recombinant inbred lines. Even though the coverage of sequence reads throughout the genome was not enough (only 0.02× coverage per line), their method provided useful results. However, this method can only be applied with cultivars whose genome sequences have been decoded, and that makes it difficult to apply their approach with a large set of independent lines or cultivars. To overcome this limitation, we performed a two-step analysis, with genome-wide SNP detection followed by array-based genome-wide SNP typing. Based on the relatively high sequencing depth, we detected a large number of SNPs between two cultivars (Nipponbare and Koshihikari) that are representative of the population being analyzed. We then developed a typing array consisting of 1917 SNP sites, and applied it to 151 representative cultivars from Japanese rice breeding history over the past 150 years (additional file 5). Even though the SNP density was insufficient in some regions of the chromosomes, we successfully defined distinct haplotypes.

Definition of the pedigree haplotypes enabled us to discriminate between almost all of the 151 cultivars, and to thereby gain insights into the changes in genome composition that have occurred during the history of modern rice breeding. Our analysis revealed that these breeding practices have clearly simplified the genome composition. Furthermore, we clearly demonstrated that the Koshihikari genome is dominant in certain regions of the chromosomes in the most recently bred cultivars. This dominance by the Koshihikari genome is not surprising, because Koshihikari has frequently been used as a parental line during cultivar development (additional file 5, Figure 3B). This information will be useful to guide future cross-breeding using recent Japanese cultivars, since it will allow breeders to avoid redundant haplotypes in crossing designs and will facilitate the selection of genotypes in the progeny of each cross combination.

We successfully identified 18 consensus haplotype blocks longer than 1 Mb in Koshihikari, Hitomebore, Akitakomachi, and Hinohikari (Figure 3C), which together account for about 65% of the total area of rice cultivation in Japan [34]. Furthermore, these haplotype blocks could be assigned to genomic regions of the ancestral landraces. These highly conserved regions are the consequence of selection during modern rice breeding, and may be an essential identifying characteristic of recent Japanese rice cultivars. Currently, we have not yet developed any annotations for these haplotype blocks. From a biological point of view, it will be necessary to clarify the relationship between haplotype conservation and recombination frequency or physical structure of chromosomes. From a rice breeding point of view, it will be necessary to clarify the association between particular haplotypes and phenotypic differences. We have recently developed reciprocal chromosomal-segment-substitution lines between Nipponbare and Koshihikari [35]. These plant materials will be invaluable for the genetic analysis of phenotypic traits that are of economic interest.

### Changes in haplotype diversity during modern rice breeding

It has been reported that genetic diversity, based on the estimated genetic distances among several rice ecotypes, is decreasing in modern rice cultivars [7]. We clearly demonstrated that the haplotype number has been decreased by breeding practices (Figure 4), supporting this idea. In barley, Rostoks et al. [25] analyzed chromosomal haplotype diversity using 1524 SNPs based on genome-wide expressed sequence tag information. They suggested that chromosome 6H is highly diverse throughout the cultivar group. In rice, chromosome 2 showed a higher level of diversity than the other chromosomes (Figure 4 and additional file 9). Interestingly, rice chromosome 2 has a syntenic relationship with barley chromosome 6H. There is limited information on the association between genes located in those genomic regions and phenotypic variation in traits of agronomic or economic interest. Thus, it is difficult to speculate about the source of this high level of diversity. This observation may nonetheless provide a new opportunity to investigate whether the same form of selective sweep during breeding has occurred in two or more related crop species such as rice and barley.

Genetic diversity was relatively low (haplotype number <0.1) in several chromosomal regions in all three groups, such as in 2.6 Mb on chromosome 1, in 4.2 Mb on chromosome 4, in 23.5 Mb on chromosome 7, in 15.2 Mb on chromosome 8, in 6.9 Mb on chromosome 11, and in 6.8 Mb on chromosome 12 (Figure 4). This suggests that there may have been genetic bottlenecks during differentiation of the Japanese landraces. It will be interesting to learn whether this low level of genetic diversity is present in those genomic regions in other *japonica *rice accessions. It would also be interesting to perform new introgression in these regions from the genomes of distantly related cultivars during breeding. In contrast, relatively highly variable regions (haplotype number > 0.4) were identified in 19.5 Mb on chromosome 10 and in 2.2 Mb on chromosome 11. Because of their high variability, these regions are not likely to have been adversely affected by selection pressure during breeding. We therefore hypothesize that there are no genes of economic value in these chromosomal regions. Alternatively, intense recombination might have occurred in these chromosomal regions during breeding.

The number of haplotypes may have decreased from Group 2 (older) to Group 3 (more recent) in chromosomal regions such as those measuring 0.9 to 1.9 Mb on chromosome 6 and 7.6 to 12.5 Mb on chromosome 12. This may be an example of decreasing genetic diversity in a particular chromosomal region as a result of breeding. This decrease might be due to the change in breeding strategy from a focus on high yield to a focus on grain and cooking quality. In fact, the short arm of chromosome 6 contains major QTLs related to flowering time [36] and the *waxy *gene, which may contribute to grain quality [37-39], and several QTLs that control grain quality are located in the middle of chromosome 12 (Gramene QTL Database, http://www.gramene.org/qtl/).

We detected regions in which the number of haplotypes increased from Group 1 to Group 3, such as in 29.8 to 33.8 Mb on chromosome 1, in 0.7 to 2.2 Mb on chromosome 3, and in 19.4 to 22.7 Mb on chromosome 12, suggesting that a new haplotype block might have been created by selection during breeding. One possible explanation is that this occurred by strong selection of two loci that become tightly linked during the repulsion phase. For example, major QTLs for eating quality (stickiness, hardness, and the appearance of cooked rice) have been mapped in a region of 0.7 to 2.2 Mb on chromosome 3 [40-42]. In addition, a major QTL related to germination ability at low temperature (*qLTG-3-1*) was identified in this region [43]. It therefore seems reasonable to hypothesize that the new haplotype was created as a result of selection for a combination of eating quality and germination ability. Further analysis will be required to prove that this type of strong selection pressure occurred during rice breeding.

### Toward next-generation breeding based on haplotype selection

The haplotypes of modern rice cultivars are the results of combinations of haplotypes inherited from ancestral landraces. Therefore, some of the haplotype blocks that remain in Group 3 might have been selected by breeders, either consciously or unconsciously. Once we reveal the relationships between the haplotype blocks and phenotypic changes in future research, it should become possible to develop ideal combinations of haplotype blocks (ideotypes). Based on the densely mapped SNP information from the present study, it will be possible to design disruption and reconstruction of existing consensus haplotype blocks [44] to generate novel haplotype blocks with new variations (i.e., to perform haplotype shuffling). On the other hand, excessive inbreeding has generally resulted in homogenization of the genome, thereby decreasing genetic diversity and creating a more uniform genome pattern in autogamous crop plants such as rice and wheat. To enhance the potential utility of the haplotype information revealed in this study, genome or haplotype shuffling should be carefully considered in future rice breeding.

To facilitate this process, the introduction of new approaches in breeding, such as recurrent selection to generate dynamic whole-genome recombination [45] and genomic selection [46,47] will be needed. In these new approaches, genome-wide SNP discovery and whole-genome SNP typing will be indispensable tools. Our results in the present study will therefore open the door for next-generation breeding in rice.

## Conclusions

Detection of genome-wide SNPs by both high-throughput sequencer and typing array made it possible to evaluate genomic composition of genetically related rice varieties. With the aid of their pedigree information, we clarified the dynamics of chromosome recombination during the historical rice breeding process. We also found several genomic regions of decreasing diversity which might be caused by a recent human selection in rice breeding. The definition of pedigree haplotypes by means of genome-wide SNPs analysis will facilitate next-generation breeding of rice and other crops.

## Methods

### Mapping Solexa reads to the reference genome

The genomic DNA of *Oryza sativa *L. cv. Koshihikari (Rice genome resource center, NIAS, http://www.rgrc.dna.affrc.go.jp/) was used for 32-bp single-read templates using the Solexa genome analyzer. After removing reads that showed high homology with the rice organelle genome (*O. sativa *ssp. *japonica *group chloroplasts and Nipponbare mitochondria, with DDBJ accession numbers of X15901 and DQ167400, respectively) using version 2.2.10 of the BLASTN software [48], we obtained approximately 270 million valid reads, corresponding to 8.9 Gb, which amounts to about 23× the rice genome. We selected reads that were uniquely mapped in the Nipponbare genome (Pseudomolecules build 4.0, http://rgp.dna.affrc.go.jp/E/IRGSP/Build4/build4.html) using ELAND (optional software for the Solexa pipeline system), allowing mismatches of up to 2 bp between a Koshihikari read and the corresponding Nipponbare sequence. We constructed consensus sequences for Koshihikari using version 0.6.6 of the MAQ software [49]. Coverage of Koshihikari in the Nipponbare genome was defined as the proportion of the bases in the reads (consensus sequences) of Koshihikari that aligned with those in the Nipponbare genome. Unidentified bases (Ns) in the Nipponbare genome (2.6%) were regarded as uncovered regions. To clarify the details of the regions of the Nipponbare genome not covered by the Koshihikari consensus sequences, we compared such regions with the TIGR plant repeat database of transposons and rDNAs [50] and with the rice whole-genome annotation (WhoGA; http://rgp.dna.affrc.go.jp/whoga/index.html.en). When the uncovered region was shorter than 30 bp, we additionally performed a BLASTN search filtered according to a homology match of >96% to confirm whether these sequences represented duplication within the Nipponbare genome or other structural changes, such as multiple SNPs or insertion-deletions (InDels). Pseudomolecules and short reads of the Koshihikari genome have been submitted to DDBJ http://www.ddbj.nig.ac.jp/index-e.html) under accession nos. [DDBJ: DG000025 to DDBJ: DG000036 and DDBJ: DRA000010 (ftp://ftp.ddbj.nig.ac.jp/ddbj_database/dra/DRA000010/)].

### Detection of genome-wide SNPs between Japanese cultivars

Genome-wide SNPs between Koshihikari and Nipponbare were detected using the MAQ software [49]. The score for consensus quality, which is an index of the depth and accuracy of the flanking sequences identified by MAQ, was set at >30 for SNP identification. To confirm whether the sequencing depth of the output Solexa reads was sufficient for genome-wide detection of inherent SNPs, we simulated the relationship between sequencing depth and the change in the number of detected SNPs. Mapped reads that were produced at a depth of 15.7× were randomly eliminated to produce adjusted sequencing depths of 10×, 5×, 2×, and 1×. The number of SNPs detected by MAQ was calculated at each of these depths. To compare detected SNPs with annotated gene structures in the rice genome, we integrated the physical positions of Koshihikari contigs, including SNP information, into both the annotated RAP2 Nipponbare full-length respective cDNAs [26] and the human curated non-redundant protein data (WhoGA; http://rgp.dna.affrc.go.jp/whoga/index.html.en) using the Generic Genome Browser (GBrowse [51]; http://koshigenome.dna.affrc.go.jp/).

### Array analysis

The SNPs used for genotyping were selected from the candidate SNPs between the Nipponbare and Koshihikari genome sequences. These SNPs were selected at a spacing of 100 to 200 kb, and their adaptability to the Illumina (San Diego, CA, USA) Golden Gate detection system was scored using the Illumina online scoring system https://icom.illumina.com. After scoring of the SNPs and their neighboring sequences, SNPs with a score higher than 0.4 were selected to design 768-plex or 384-plex SNPs for the Illumina GoldenGate BeadArray technology platform.

Based on known pedigrees and development dates, we selected 151 representative Japanese cultivars of *Oryza sativa *L. ssp. *japonica *that covered the entire modern history of rice breeding in Japan (additional file 3). We categorized these accessions into three groups based on the years when they were developed (see Results for details). Total DNA was extracted from a piece of a leaf blade from each accession [52], and we used 5 μL of 50 ng/μL DNA in the SNP analysis. These SNPs were detected using the Illumina Bead Station 500G system. All experimental procedures for the SNP typing followed the manufacturer's instructions. We discarded SNPs with: (1) no signals in any genome samples, (2) no signals in the Nipponbare or Koshihikari genomes, (3) no signals in more than 10% of the genome samples, (4) no polymorphism, and (5) more than 10% of the signals recognized as heterozygous. Based on these screening criteria, we retained 1917 SNPs http://koshigenome.dna.affrc.go.jp/ out of 2688 initially designed SNPs (768-plex × 3 and 384-plex × 1) and used them in our subsequent analysis.

### Visualization of the genotypes

We automatically visualized the genotypes of the 151 Japanese rice cultivars using a proprietary program written in Microsoft Visual Basic for Applications (VBA) in Microsoft Excel 2007 as follows. The VBA program translated the SNP information and the physical position of the SNP into a color and length (number of cells) in the Excel spreadsheet to provide a graphical representation of the genotype. The colors used in the graphical genotype representation for each cultivar were based on a comparison with the genotypes of Koshihikari and Nipponbare. If the genotype equaled that of Koshihikari, it was colored blue. If the genotype equaled that of Nipponbare, it was colored yellow. If the genotype was neither Koshihikari nor Nipponbare, it was colored dark green for heterozygous and grey for missing data. The borders of each cell in the graphical genotype representation represent points intermediate between the physical positions of two adjacent SNPs. The cell width (number of cells) used to represent the region size in the graphical representations was 13 pixels per 1 Mb. Regions of the graphical genotype smaller than about 77 kb (= 1 Mb/13 pixels) were represented as a single pixel, since this is the smallest display size in Excel.

### Identification of the Koshihikari pedigree haplotype blocks

The genotype constitutions of the ancestors of Koshihikari (additional file 6) were compared with the Koshihikari genotype, and identical haplotype blocks were extracted when the region of the genotype blocks that were identical to the Koshihikari genotype was 500 kb or longer. Haplotype blocks that successfully traced pedigrees to an ancestral cultivar were defined as ancestral pedigree haplotype blocks. The successful haplotype blocks obtained for three cultivars (Hitomebore, Akitakomachi, and Hinohikari; additional files 3 and 6), which are the second, third, and fourth most-grown cultivars in Japan and which were developed by crossing with Koshihikari, were also defined as ancestral pedigree haplotype blocks. Successful haplotype blocks common to Hitomebore, Akitakomachi, and Hinohikari were defined as "consensus haplotype blocks".

### The haplotype diversity index

To determine an appropriate size for the sliding window that should be used for estimation of the haplotype diversity index, we calculated normalized pairwise disequilibria values (*D*'; [53]) between each pair of SNPs within 5000 kb of each other on rice chromosome 1 (additional file 7). We used only biallelic and homozygous SNPs for the estimation of linkage disequilibrium (LD). We combined the *D*' data into 200-kb distance intervals (determined by dividing the ca. 382 Mb rice genome by the 1917 SNPs used in our analysis) to reduce the influence of outliers and to obtain a median value for a better visual description of the decay in LD as a function of distance. We selected four distances as LD decay points for simulation of the size of the sliding window: 0.4, 1, 2, and 3 Mb, which correspond to window sizes of 2, 5, 10, and 15 SNPs, respectively, based on the assumed mean distance (200 kb) between adjacent SNPs in the rice genome. The haplotype diversity index was simulated only for 38 cultivars belonging to Group 1, because this was the oldest group of cultivars, and would therefore show the fewest effects of artificial selection on the haplotype diversity. The index was calculated by dividing the number of haplotypes into the number of cultivars in this group (i.e., 38). Haplotype windows covering 2, 5, 10, and 15 SNPs were moved from the short arm of chromosome 1 to the long arm one SNP at a time, and we calculated the number of haplotypes in each window (additional file 8). Both the LD graph and the results of the simulation indicated that a 2-Mb window (10 SNPs) was the most appropriate size for estimation of the haplotype diversity index. Using this result, we estimated the haplotype diversity index for each of the three groups of cultivars in additional file 9 for each chromosome. To compare the indices among the three groups of cultivars, we used the Friedman test, as implemented in the R statistical software http://cran.r-project.org/.

## Authors' contributions

TY and HN performed most of the experiments. MY initiated and coordinated the study. TY, HN, and MY wrote the manuscript. HN performed the SNP discovery. JY performed haplotype and diversity analysis. MN and TS contributed to the SNP typing of the rice accessions. KE provided the core rice collection and performed the SNP analysis. All authors discussed the results and commented on the manuscript. All authors read and approved the final manuscript.

## Supplementary Material

Additional file 1 **Sequencing depth of the Koshihikari short reads generated using the Solexa Genome Analyzer with reference to the Nipponbare genome**. The x-axis shows the physical distance along the rice reference genome (Nipponbare). Red lines indicate regions in which the Koshihikari short reads could not be mapped at all owing to low reliability of the Nipponbare reference sequence (i.e., regions containing more than 50% unidentified bases, Ns). The y-axis shows the sequencing depth, which represents the mean number of reads mapped in 100-kb windows. The mean total sequencing depth was 15.7× the genome (Table 1). Click here for file

Additional file 2 **Relationships among the coverage of the rice genome by short read contigs and the number of SNPs detected as a function of the sequencing depth**. White bars correspond to the left vertical scale (% coverage of the genome by the assembled short read contigs) and the black line corresponds to the right vertical scale (number of SNPs detected). Click here for file

Additional file 3 List of the *Oryza sativa *ssp. *japonica *cultivars used in the single-nucleotide polymorphism (SNP) analysis. (Group 1, late 1800s to about 1921; Group 2, 1931 to 1974; Group 3, 1975 to 2005). Click here for file

Additional file 4 **Population structure analysis plots with three K values (K = 2, 3, 4) using STRUCTURE program **[54]. The top panel (K = 2) showed that Japanese cultivars were divided by 192 SNP genotypes of Nipponbare (Red arrow) and Koshihikari (Green arrow). This is natural result because all SNPs tested were extracted from the mapping result of Koshihikari short sequences to Nipponbare genome. The other panels show the relatively same population size of Koshihikari SNP group, but with further population subdivision within the Nipponbare SNP group from K = 3. All panels (K = 2, 3, 4) indicate that population structure was not associated with the year each cultivar released. Click here for file

Additional file 5 **Graphical representation of the genotypes of the 151 Japanese rice landraces and cultivars in additional file**3** that have been grown in the past 150 years**. To visualize the genome compositions of these cultivars, the SNP type is indicated in blue (Koshihikari type; No. *61 *in additional file 3), in yellow (Nipponbare type; *72*), in dark green (heterozygous), and in gray (missing data). The cultivar names are ordered from top to bottom by their year of development, as are the three groups (Group 1, late 1800s to about 1921; Group 2, 1931 to 1974; Group 3, 1975 to 2005). Click here for file

Additional file 6 **Pedigree diagram for Japanese rice cultivars that are ancestors or descendants of Koshihikari**. Numbers in parentheses are the serial numbers designated in additional file 3. Black, shaded, and white backgrounds represent the following varietal groups, respectively: Group 1 (landraces developed before 1921), Group 2 (cultivars developed from 1931 to 1974), and Group 3 (cultivars developed from 1975 to 2005). This classification is based on the categorization of R. Yamamoto [55]. Click here for file

Additional file 7 **Median values of the estimate of linkage disequilibrium (*D'*) between SNP pairs within a 5000-kb distance on rice chromosome 1**. The median *D' *was calculated using 200-kb distances. Triangles represent the points for the simulation of the size of sliding window at distances of 0.4, 1, 2, and 3 Mb. Click here for file

Additional file 8 **Simulation results from the sliding-window haplotype analysis of cultivars in Group 1 on chromosome 1**. Changes in the haplotype diversity index are based on 2-, 5-, 10-, and 15-SNP sliding windows on the assumption of a mean distance of 200 kb between adjacent SNP pairs in the rice genome. On this basis, the window sizes of the 2-, 5-, 10-, and 15-SNP sliding windows were 0.4, 1, 2, and 3 Mb respectively. Click here for file

Additional file 9 Average number of haplotypes per 10-SNP interval. Click here for file
