# Supplementary figures and images for: Fine definition of the pedigree haplotypes of closely related rice cultivars by means of genome-wide discovery of single-nucleotide polymorphisms
Source: BMC Genomics. 2010 Apr 27;11:267. doi: 10.1186/1471-2164-11-267 (PMC2874813; doi:10.1186/1471-2164-11-267)

## Slide 1
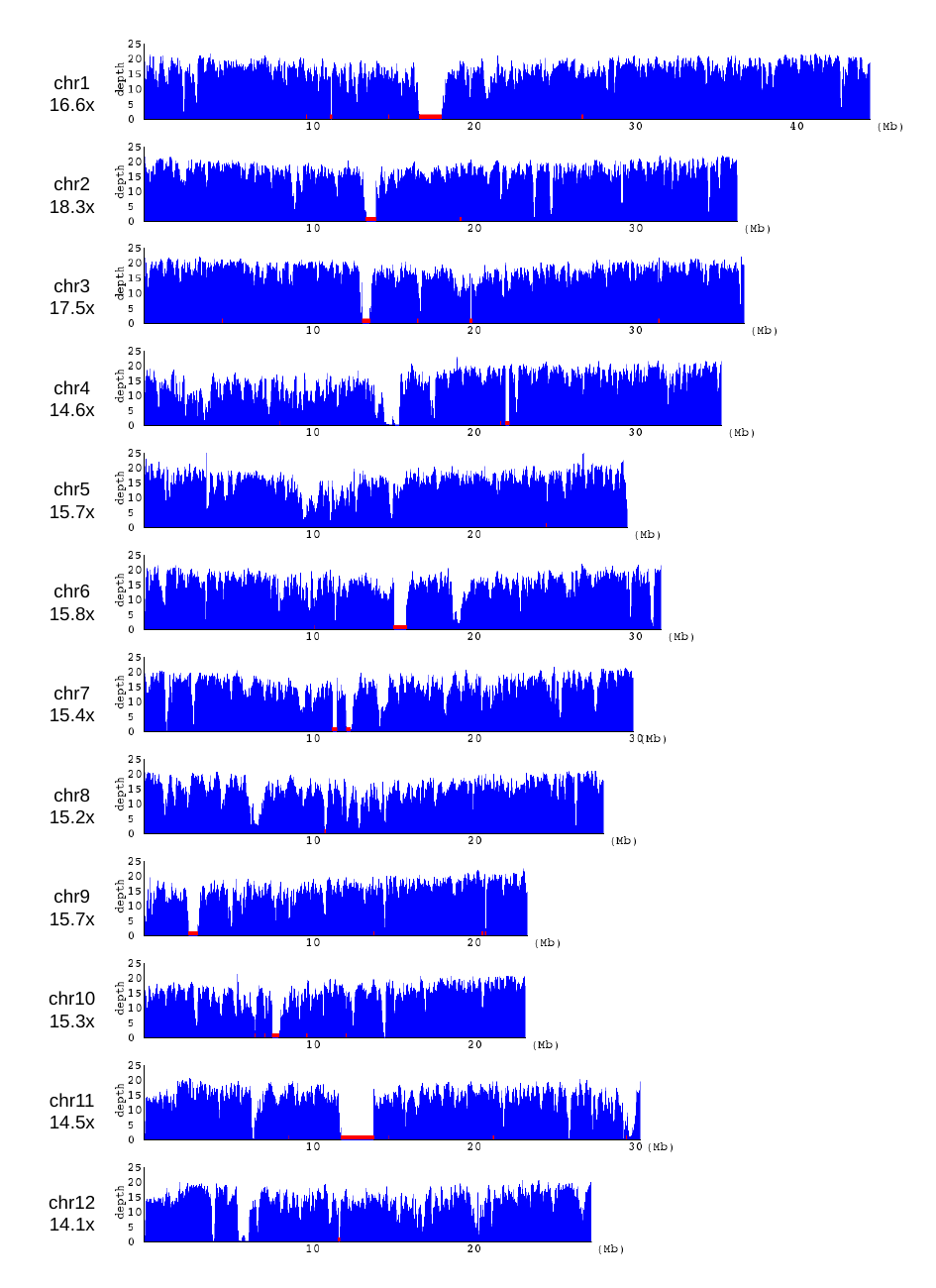

chr1
16.6x
chr2
18.3x
chr3
17.5x
chr4
14.6x
chr5
15.7x
chr6
15.8x
chr7
15.4x
chr8
15.2x
chr9
15.7x
chr10
15.3x
chr11
14.5x
chr12
14.1x

Supplement: Additional file 1 — Sequencing depth of the Koshihikari short reads generated using the Solexa Genome Analyzer with reference to the Nipponbare genome. The x-axis shows the physical distance along the rice reference genome (Nipponbare). Red lines indicate regions in which the Koshihikari short reads could not be mapped at all owing to low reliability of the Nipponbare reference sequence (i.e., regions containing more than 50% unidentified bases, Ns). The y-axis shows the sequencing depth, which represents the mean number of reads mapped in 100-kb windows. The mean total sequencing depth was 15.7× the genome (Table 1). [file 1471-2164-11-267-S1.PPT]

## Slide 1
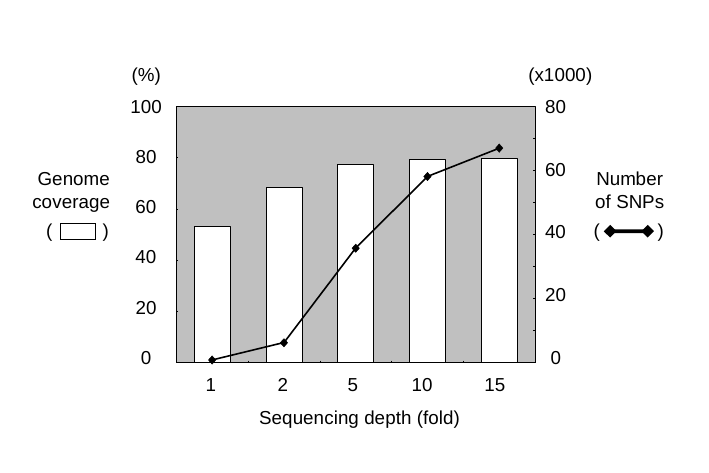

(%)
(x1000)
100
80
80
60
Genome coverage
Number of SNPs
60
(
)
(
)
40
40
20
20
0
0
1
2
5
10
15
Sequencing depth (fold)

Supplement: Additional file 2 — Relationships among the coverage of the rice genome by short read contigs and the number of SNPs detected as a function of the sequencing depth. White bars correspond to the left vertical scale (% coverage of the genome by the assembled short read contigs) and the black line corresponds to the right vertical scale (number of SNPs detected). [file 1471-2164-11-267-S2.PPT]

## Slide 1
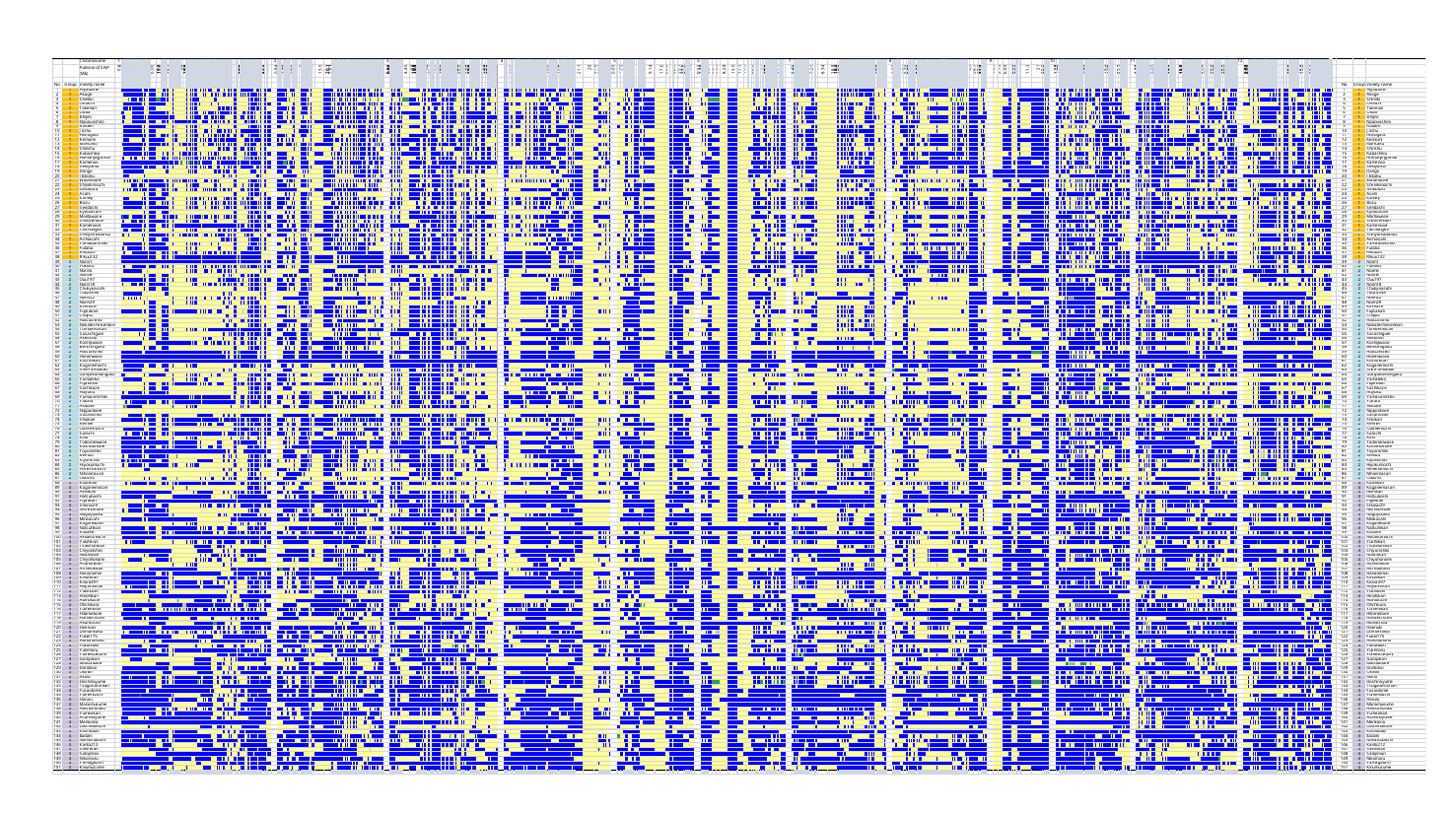

Supplement: Additional file 5 — Graphical representation of the genotypes of the 151 Japanese rice landraces and cultivars in additional file3 that have been grown in the past 150 years. To visualize the genome compositions of these cultivars, the SNP type is indicated in blue (Koshihikari type; No. 61 in additional file 3), in yellow (Nipponbare type; 72), in dark green (heterozygous), and in gray (missing data). The cultivar names are ordered from top to bottom by their year of development, as are the three groups (Group 1, late 1800s to about 1921; Group 2, 1931 to 1974; Group 3, 1975 to 2005). [file 1471-2164-11-267-S5.PPT]

## Slide 1
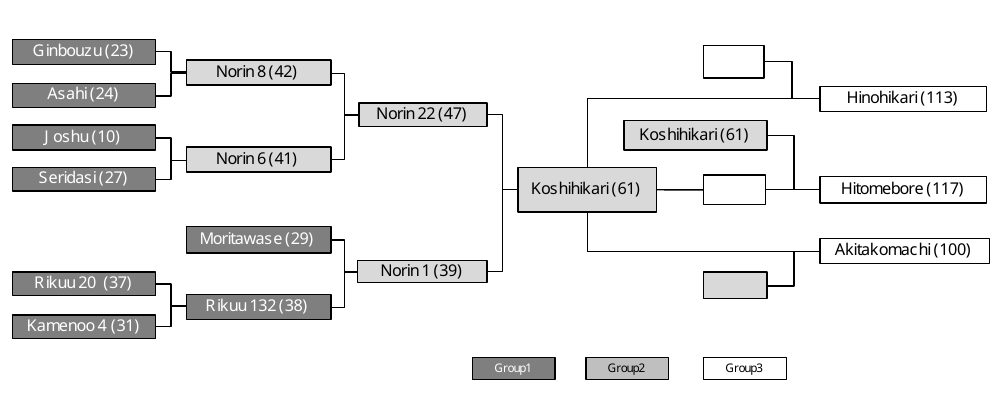

Supplement: Additional file 6 — Pedigree diagram for Japanese rice cultivars that are ancestors or descendants of Koshihikari. Numbers in parentheses are the serial numbers designated in additional file 3. Black, shaded, and white backgrounds represent the following varietal groups, respectively: Group 1 (landraces developed before 1921), Group 2 (cultivars developed from 1931 to 1974), and Group 3 (cultivars developed from 1975 to 2005). This classification is based on the categorization of R. Yamamoto [55]. [file 1471-2164-11-267-S6.PPT]

## Slide 1
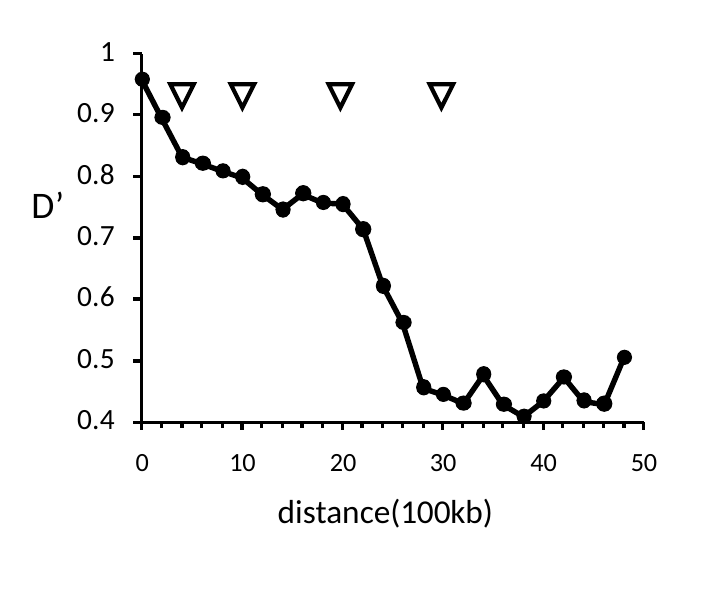

D’
distance(100kb)

Supplement: Additional file 7 — Median values of the estimate of linkage disequilibrium (D') between SNP pairs within a 5000-kb distance on rice chromosome 1. The median D' was calculated using 200-kb distances. Triangles represent the points for the simulation of the size of sliding window at distances of 0.4, 1, 2, and 3 Mb. [file 1471-2164-11-267-S7.PPT]

## Slide 1
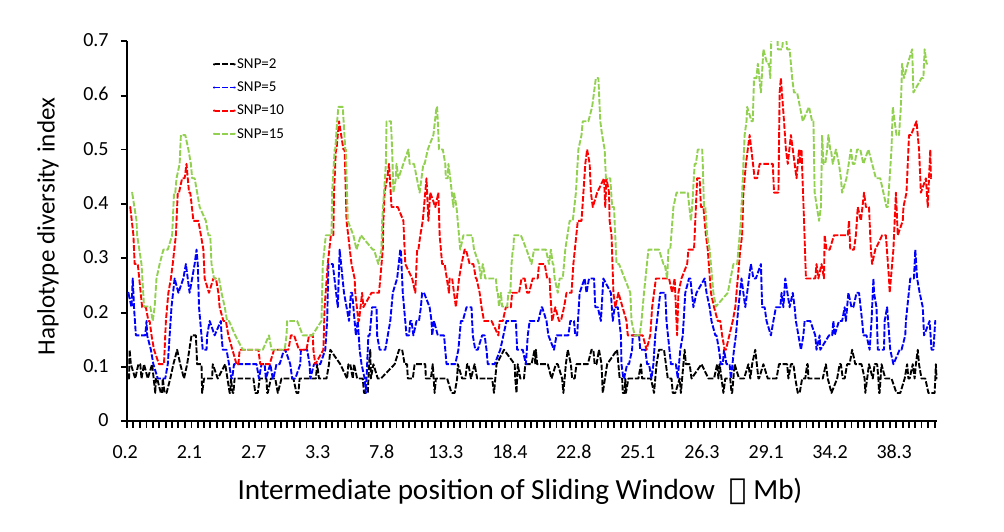

Haplotype diversity index
Intermediate position of Sliding Window （Mb)

Supplement: Additional file 8 — Simulation results from the sliding-window haplotype analysis of cultivars in Group 1 on chromosome 1. Changes in the haplotype diversity index are based on 2-, 5-, 10-, and 15-SNP sliding windows on the assumption of a mean distance of 200 kb between adjacent SNP pairs in the rice genome. On this basis, the window sizes of the 2-, 5-, 10-, and 15-SNP sliding windows were 0.4, 1, 2, and 3 Mb respectively. [file 1471-2164-11-267-S8.PPT]
