# Supplementary material for: Fine definition of the pedigree haplotypes of closely related rice cultivars by means of genome-wide discovery of single-nucleotide polymorphisms
Source: BMC Genomics. 2010 Apr 27;11:267. doi: 10.1186/1471-2164-11-267 (PMC2874813; doi:10.1186/1471-2164-11-267)
Supplement: Additional file 4 — Population structure analysis plots with three K values (K = 2, 3, 4) using STRUCTURE program [54]. The top panel (K = 2) showed that Japanese cultivars were divided by 192 SNP genotypes of Nipponbare (Red arrow) and Koshihikari (Green arrow). This is natural result because all SNPs tested were extracted from the mapping result of Koshihikari short sequences to Nipponbare genome. The other panels show the relatively same population size of Koshihikari SNP group, but with further population subdivision within the Nipponbare SNP group from K = 3. All panels (K = 2, 3, 4) indicate that population structure was not associated with the year each cultivar released. [file 1471-2164-11-267-S4.PPT]

## Slide 1
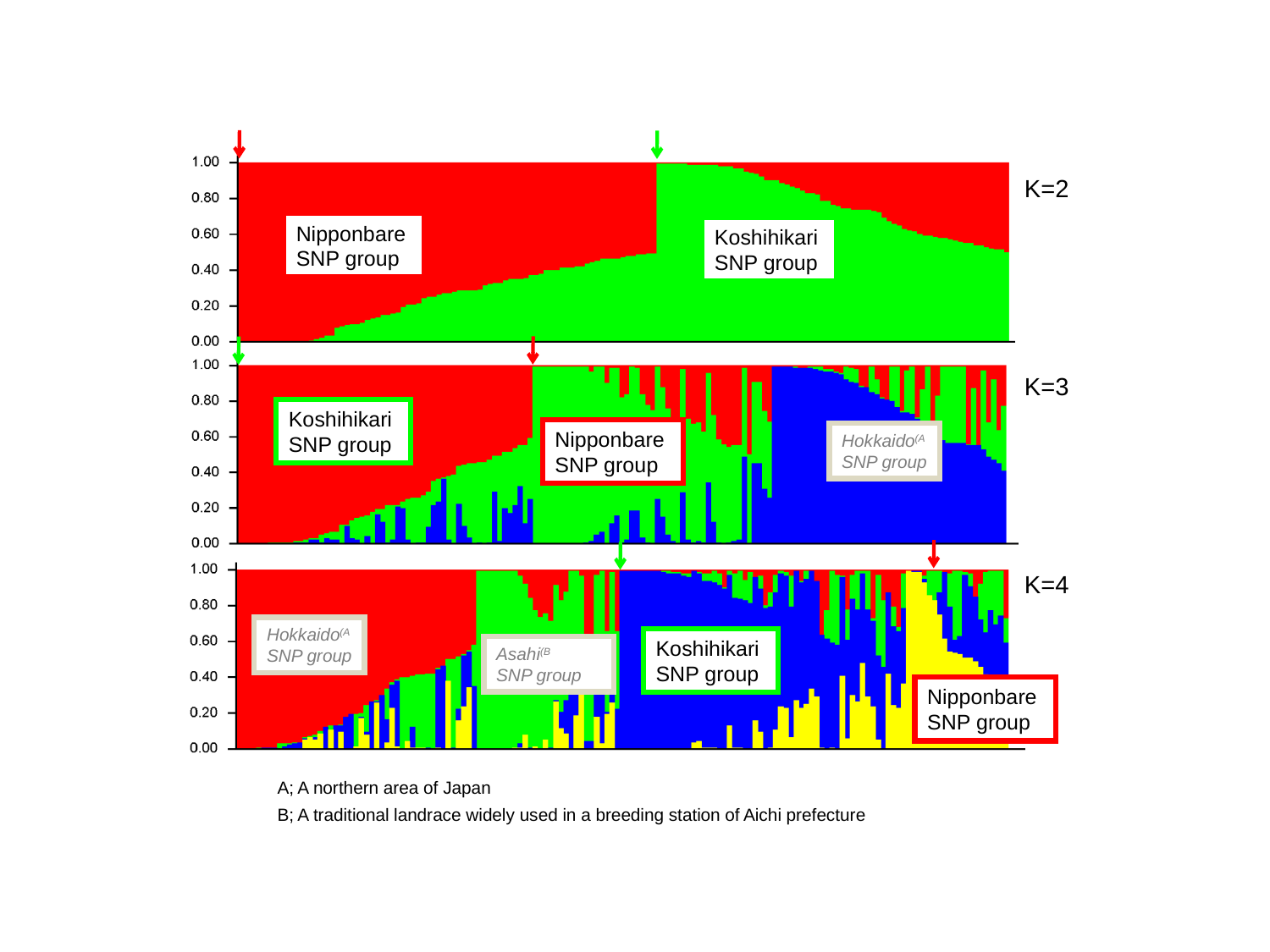

K=2
Nipponbare
SNP group
Koshihikari
SNP group
K=3
Koshihikari
SNP group
Nipponbare
SNP group
Hokkaido(A
SNP group
K=4
Hokkaido(A
SNP group
Koshihikari
SNP group
Asahi(B
SNP group
Nipponbare
SNP group
A; A northern area of Japan
B; A traditional landrace widely used in a breeding station of Aichi prefecture
